# Costs and Impacts of Scaling up Voluntary Medical Male Circumcision in Tanzania

**DOI:** 10.1371/journal.pone.0083925

**Published:** 2014-05-06

**Authors:** Veena Menon, Elizabeth Gold, Ramona Godbole, Delivette Castor, Hally Mahler, Steven Forsythe, Mariam Ally, Emmanuel Njeuhmeli

**Affiliations:** 1 HPI Costing Task Order, Washington, District of Columbia, United States of America; 2 Futures Group, Washington, District of Columbia, United States of America; 3 Maternal and Child Health Integrated Program Jhpiego, Baltimore, Maryland, United States of America; 4 United States Agency for International Development, Washington, District of Columbia, United States of America; 5 Maternal and Child Health Integrated Program Jhpiego, Dar es Salaam, Tanzania; 6 Futures Institute, Glastonbury, Connecticut, United States of America; 7 Ministry of Health and Social Welfare, Dar es Salaam, Tanzania; World Health Organization, Switzerland

## Abstract

**Background:**

Given the proven effectiveness of voluntary medical male circumcision (VMMC) in preventing the spread of HIV, Tanzania is scaling up VMMC as an HIV prevention strategy. This study will inform policymakers about the potential costs and benefits of scaling up VMMC services in Tanzania.

**Methodology:**

The analysis first assessed the unit costs of delivering VMMC at the facility level in three regions—Iringa, Kagera, and Mbeya—via three currently used VMMC service delivery models (routine, campaign, and mobile/island outreach). Subsequently, using these unit cost data estimates, the study used the Decision Makers' Program Planning Tool (DMPPT) to estimate the costs and impact of a scaled-up VMMC program.

**Results:**

Increasing VMMC could substantially reduce HIV infection. Scaling up adult VMMC to reach 87.9% coverage by 2015 would avert nearly 23,000 new adult HIV infections through 2015 and an additional 167,500 from 2016 through 2025—at an additional cost of US$253.7 million through 2015 and US$302.3 million from 2016 through 2025. Average cost per HIV infection averted would be US$11,300 during 2010–2015 and US$3,200 during 2010–2025. Scaling up VMMC in Tanzania will yield significant net benefits (benefits of treatment costs averted minus the cost of performing circumcisions) in the long run—around US$4,200 in net benefits for each infection averted.

**Conclusion:**

VMMC could have an immediate impact on HIV transmission, but the full impact on prevalence and deaths will only be apparent in the longer term because VMMC averts infections some years into the future among people who have been circumcised. Given the health and economic benefits of investing in VMMC, the scale-up of services should continue to be a central component of the national HIV prev**e**ntion strategy in Tanzania.

## Introduction

According to three randomized controlled trials, voluntary medical male circumcision (VMMC) reduces the risk of heterosexually acquired HIV infection in men by approximately 60% [1]–[3]. Thus, as part of its HIV prevention strategy, the World Health Organization (WHO) and Joint United Nations Program on HIV/AIDS (UNAIDS) recommend that VMMC programs be scaled up in countries with relatively low male circumcision prevalence and high HIV prevalence. This analysis focuses on VMMC's benefits as an HIV prevention strategy. While there would be additional benefits for prevention of other STIs, these have not been quantified in this analysis.

In 2009, the Government of Tanzania incorporated VMMC as part of its HIV prevention portfolio by concentrating efforts in areas where male circumcision prevalence was below 80% with the goal of reaching 80% in those regions. The Ministry of Health and Social Welfare identified regions with a relatively high burden of HIV and low prevalence of male circumcision for delivering VMMC services (see Table 1) [4]. As this table shows, male circumcision prevalence rose between 2007 and 2011 in six of the eight regions, decreased in one of the eight regions, and was uncertain in one region.

**Table 1:** Male HIV prevalence and male circumcision prevalence in Tanzania, 2007 and 2012.

Targeted Regions	HIV Prevalence		Male Circumcision Prevalence	
	2007	2012	2007	2012
**Iringa:**	15.7%	-	37.7%	59.7%^a^^,^ b
Iringa	-	9.1%	-	-
Njombe	-	14.8%	-	49.2%
**Kagera**	3.4%	4.8%	26.4%	38.9%
**Mbeya**	9.2%	9.0%	34.4%	37.9%
**Mara**	7.7%	4.5%	89.0%	87.6%
**Mwanza**	5.5%	4.2%	54.1%	63.8%
**Rukwa:**	4.9%	-	31.4%	-
Rukwa	-	6.2%	-	27.5%
Katavi	-	5.9%	-	44.3%
**Shinyanga:**	7.4%	-	26.5%	-
Shinyanga	-	7.4%	-	32.1%^c^
Geita	-	4.7%	-	41.0%
Simiyu	-	3.6%	-	30.4%
**Tabora**	6.4%	-	42.8%	55.6%

**References:**

1. 2007 Tanzania Demographic and Health Survey, Tanzania Commission for AIDS (TACAIDS), Zanzibar AIDS Commission (ZAC), National Bureau of Statistics (NBS), Office of the Chief Government Statistician (OCGS), and ICF International 2013.

2. Tanzania HIV/AIDS and Malaria Indicator Survey 2011–12. Dar es Salaam, Tanzania: TACAIDS, ZAC, NBS, OCGS, and ICF International.

a In 2012, Iringa was subdivided up into 2 regions: Iringa and Njombe.

b Based on the Iringa's boundaries in 2007, male circumcision prevalence was 38 percent.

c In 2012, Shinyanga was subdivided into 3 regions: Shinyana, Geita, and Simiyu.

**Source: Tanzania DMPPT Impact Model.**

Male circumcision prevalence varies across geographical areas and ranges from a low of 27.5 percent in Rukwato a high of −100 percent in Kusini Pemba. The eight regions targeted for scale-up include Iringa, Kagera, Mara, Mbeya, Mwanza, Rukwa, Shinyanga, and Tabora. Table 1 outlines both male HIV prevalence and male circumcision prevalence for each region. The delivery of VMMC services is integrated within the existing public and private health delivery system, comprising referral, specialized, regional, and district hospitals and primary health facilities (health centers).

The delivery of VMMC services in Tanzania incorporates many of the WHO's recommendations for increasing program efficiency, as outlined in its “Models for Optimizing the Volume and Efficiency of Male Circumcision Services.” These recommended strategies include optimizing the use of facility space (using multiple surgical beds in one room), maximizing staff time by task shifting and task sharing, using VMMC kits that include disposable consumables and sets of reusable or disposable surgical instruments, and employing a multi-disciplinary team of trained and certified providers drawn from available service providers in health facilities to provide VMMC services.

This study estimated the unit cost of providing VMMC services through various service delivery models in Tanzania—routine, campaign, and mobile/island outreach—as well as the cost and impact of scaling up adult VMMC to reach 80% of males in regions where current coverage is less than 80%. The findings will assist health ministries as well as donors in planning and allocating resources for VMMC implementation.

## Methods

### Model

The study used the Decision Makers' Program Planning Tool (DMPPT) [5] to estimate the cost of scaling up VMMC and the impact on infections averted in 2010/2011. The tool was developed by the US Agency for International Development | Health Policy Initiative, Task Order 1 in collaboration with UNAIDS to enable decision-makers to understand the potential cost and impact of various options for scaling up male circumcision services.

The age range used for the analysis was 15–49 years old and was based on two factors: (1) this is the age range the Demographic and Health Surveys (DHS) uses, and (2) these are the ages when sexual transmission of HIV is most likely to occur. However, in Tanzania, VMMC scale-up has largely been received by adolescent boys between ages 10 and 14. Therefore, for the purpose of the impact analysis, the model was adapted to include males between the age of 10 and 14.

In 2010, a total of twelve sites were providing VMMC services in three regions. All these sites were included in this analysis. Data from facilities was collected by interviewing staff at facilities and within the national government. Data collectors did not review any individual client records and did not conduct any interviews directly with clients. The Tanzania Ministry of Health granted permission to use the data, approved the protocol and provided clearance for this manuscript to be submitted for peer review publication. Respondents were aware that the data would be used for the purpose of conducting costing research and provided verbal consent to the data collectors. The team obtained IRB approvals in the United States and Tanzania.

### Cost Data

In its “Best Practices for VMMC Site Operations,” the U.S. President's Emergency Plan for AIDS Relief (PEPFAR), outlines various models and staffing options for delivering VMMC services that address specific needs of the community and the VMMC program [6]. For this study, unit costs were derived for three service delivery models currently used in Tanzania's priority regions where VMMC is being scaled up:

#### Routine

Comprises fixed sites in the existing public and private health delivery system (including district, regional, and referral hospitals and health centers).

#### Campaign

Comprises both fixed and outreach sites as part of high-volume service delivery events. The staff for these campaigns comes largely from existing, dedicated VMMC facilities to meet the demand for VMMC services.

#### Mobile/island outreach

Comprises a team of VMMC providers who offer mobile services (being coordinated from a fixed site located at a hospital) in the hard-to-reach islands of Lake Victoria, which have traditionally lacked access to many health interventions.

To calculate the unit cost of providing VMMC, human resources, utilization, and financial data were collected at 12 sites representing each service delivery model in Iringa, Kagera, and Mbeya, which were among the first of the target regions for VMMC implementation. Data were collected for 2010 and 2011 through database review and interviews were conducted with facility managers and medical officers. Of the 12 sites, two sites offered VMMC as part of both a campaign and routine service delivery model. As a result, a total of 14 unit costs were estimated (11 routine, 2 campaign, and 1 mobile/island outreach).

Unit costs were derived using an ingredients approach, whereby all the inputs were listed and their contribution to the overall cost was then quantified. The costing took into account both the direct costs (consumables, non-consumable supplies, and personnel) and indirect costs (capital, maintenance and utility, support personnel, and management and supervision). Consumables include drugs and supplies, while non-consumable supplies include items such as surgical gowns, sterile drapes, and surgical equipment used during pre- and post-circumcision. Maintenance costs included expenses for renovating new sites for VMMC provision, as well as emergency vehicle maintenance. Utilities included fuel consumption, transport costs, and electricity costs. Overhead costs, such as equipment, utilities, transport, maintenance, and support costs, were calculated based on the relative share of VMMC compared to the total facility workload. Although the protocol was modified to include the collection of cost data related to demand creation and waste management, the data were not available and thus excluded from the calculation of unit costs. Costs incurred by VMMC clients (e.g., client travel costs, clients' opportunity cost of travel time, their opportunity cost of post-operative healing time) were also excluded from the analysis.

There were important differences among the service delivery models, such as the number of VMMC providers involved in service provision, number and utilization of surgical bays, and number of VMMCs performed.

The weighted unit cost for routine, campaign, and mobile/island outreach sites in each region were used to generate an average weighted unit cost by type of service delivery model and a weighted overall unit cost for VMMC in Tanzania.

### Cost and Impact Analysis

This paper analyses the cost and impact of scaling up VMMC to reach 80% of males (ages 10–49) in eight Tanzanian regions where current coverage is less than 80%, while maintaining VMMC coverage at existing levels in regions where male circumcision coverage is above 80%. According to the 2010 Tanzania DHS, countrywide, male circumcision coverage was 72.3% among males between 15 and 49 years old. By assuming coverage increases to 80% in regions with low male circumcision prevalence, while maintaining current coverage of at least 80% in other regions, this analysis uses an overall country target of 87.9% by 2015. Future costs and benefits were discounted to 2010 at 3% annually, as has been used in previous VMMC costing studies [7], [8].

## Results

### Unit Costs of VMMC by Service Delivery Model

#### Routine service delivery

Routine service delivery models were in place in fixed sites within health facilities in all three regions. For routine service delivery, Mbeya reported the largest number of VMMCs performed annually (10,568), followed by Iringa (5,244) and Kagera (4,057). The weighted average cost of performing VMMC at a routine site in Iringa was US$48.28 (Tshs. 70,923.32; Exchange rate US$1 = Tsh 1469 (2010)). The costs per VMMC in Mbeya and Kagera were estimated at US$47.51 (Tshs. 69,792.19) and US$36.07 (Tshs. 52,986.83), respectively (see Table 2). The overall unit cost for routine sites in Tanzania was estimated at US$45.38 based on a weighted average of unit costs in the three regions according to number of VMMCs performed.

**Table 2:** 

A) VMMC unit costs by region and service delivery model: routine/fixed sites.								
	Iringa		Mbeya		Kagera		Tanzania Average	
Number of VMMCs	5,244		10,568		4,057		19,869^a^	
	Cost (US$)	%	Cost (US$)	%	Cost (US$)	%	Cost (US$)	%
**Direct costs**								
**Consumables costs**	11.04	23%	16.7	35%	16.79	47%	15.22	34%
**Non-consumable supplies costs**	0.13	0%	0.07	0%	0.1	0%	0.09	0%
**Personnel costs**	18.57	38%	18.06	38%	12.16	34%	16.99	37%
**Training costs**	12.46	26%	10.35	22%	5.03	14%	9.82	22%
***Sub-total***	*42.2*	*87%*	*45.18*	*95%*	*34.07*	*94%*	*42.12*	*93%*
**Indirect costs**								
**Capital costs**	1.14	2%	1.57	3%	1.42	4%	1.43	3%
**Maintenance and utility costs**	3.99	8%	0.16	0%	0.36	1%	1.21	3%
**Support personnel costs**	0.36	1%	0.57	1%	0.22	1%	0.44	1%
**Management and supervision costs**	0.58	1%	0.03	0%	0.01	0%	0.17	0%
***Sub-total***	*6.08*	*13%*	*2.32*	*5%*	*2.01*	*6%*	*3.25*	*7%*
**Total**	**48.28**	**100%**	**47.51**	**100%**	**36.07**	**100%**	**45.38**	**100%**

B) VMMC unit costs by region and service delivery model: campaign services in fixed sites.								
	Iringa		Mbeya		Kagera		Tanzania Average	
Number of VMMCs	12,923		N/A		214		13,137^a^	
	Cost (US$)	%	Cost (US$)	%	Cost (US$)	%	Cost (US$)	%
**Direct costs**								
**Consumables costs**	11	24%	n/a		16.7	18%	11.09	24%
**Non-consumable supplies costs**	0.13	0%	n/a		0.1	0%	0.13	0%
**Personnel costs**	20.72	46%	n/a		10.8	12%	20.56	45%
**Training costs**	4.79	11%	n/a		62.42	67%	5.73	12%
***Sub-total***	*36.64*	*81%*	n/a		*90.01*	*97%*	*37.51*	*82%*
**Indirect costs**								
**Capital costs**	1.64	4%	n/a		2.66	3%	1.66	4%
**Maintenance and utility costs**	3.99	9%	n/a		0.17	0%	3.93	9%
**Support personnel costs**	1.98	4%	n/a		0.18	0%	1.95	4%
**Management and supervision costs**	0.95	2%	n/a		0.01	0%	0.93	2%
***Sub-total***	*8.57*	*19%*	n/a		*3.03*	*3%*	*8.48*	*18%*
**Total**	**45.21**	**100%**	n/a		**93.05**	**100%**	**45.98**	**100%**

C) VMMC unit costs by region and service delivery model: mobile/island sites.								
	Iringa		Mbeya		Kagera		Tanzania Average	
Number of VMMCs	n/a		n/a		415		415^a^	
	Cost (US$)	%	Cost (US$)	%	Cost (US$)	%	Cost (US$)	%
**Direct costs**								
**Consumables costs**	n/a		n/a		16.87	13%	16.87	13%
**Non-consumable supplies costs**	n/a		n/a		0.1	0%	0.1	0%
**Personnel costs**	n/a		n/a		14.8	12%	14.8	12%
**Training costs**	n/a		n/a		14.28	11%	14.28	11%
***Sub-total***	n/a		n/a		*46.05*	*36%*	*46.05*	*36%*
**Indirect costs**								
**Capital costs**	n/a		n/a		39.74	31%	39.74	31%
**Maintenance and utility costs**	n/a		n/a		38.38	30%	38.38	30%
**Support personnel costs**	n/a		n/a		4.32	3%	4.32	3%
**Management and supervision costs**	n/a		n/a		0.11	0%	0.11	0%
***Sub-total***	n/a		n/a		*82.55*	*64%*	*82.55*	*64%*
**Total**	n/a		n/a		**128.6**	**100%**	**128.6**	**100%**

a The number of VMMCs presented is a total figure for all sites sampled.

**Source: Tanzania DMPPT Impact Model.**

Personnel costs accounted for the single largest contribution to the unit costs in all three provinces—about 38% of the total costs in Iringa and Mbeya and 34% in Kagera. Personnel costs per VMMC were significantly higher in Iringa and Mbeya (US$18.57 and US US$18.06, respectively) compared with Kagera (US$12.16). At the time of this study, fixed government scale salaries for Kagera service providers may have helped to reduce costs, as compared to Iringa and Mbeya, where service providers were paid overtime allowances for after-hours work. Consumable costs accounted for 23%, 35%, and 47% in Iringa, Mbeya, and Kagera, respectively, and were the second largest contributor to the total unit cost.

#### Campaign service intensity model

Table 2 summarizes the unit costs for VMMC performed under the campaign model in Iringa and Kagera. Mbeya did not conduct VMMC campaigns during the study period. In Iringa, campaigns were completed in June/July 2010 and in December 2010, with a total of 12,923 VMMCs performed. In Kagera, only one campaign was conducted; it was completed in March 2010, with 214 VMMCs performed. The relatively high number of VMMCs completed during the Iringa campaigns was due to a combination of factors, including strong support from the local leadership, an effective mobilization strategy that promoted local ownership, a supportive VMMC task force to oversee the planning and implementation of the campaigns, and the training of 45 additional providers and 20 counselors. The cost of delivery for one VMMC via a campaign model in Iringa was estimated at US$45.21, while the cost in Kagera was much higher at US$93.05. The higher cost in Kagera is due in large part to the small number of circumcisions being performed (214 over a period of a year, as compared to 12,923 performed within a year in Iringa). The weighted average unit cost for campaign sites across Tanzania was estimated at US$45.98.

Personnel costs accounted for the largest share (46%) of the unit cost in Iringa, while training costs accounted for the largest expense (67%) in Kagera. This variation between the two regions largely stems from the vast difference in the number of VMMCs conducted. For example, the high cost of personnel in Iringa is largely attributable to the overtime paid to VMMC providers and the large number of VMMC personnel involved in service provision during the campaign period. In Kagera, the high training costs are due to the large number of VMMC staff who underwent the training relative to the actual number of VMMCs performed during the campaign period (a total 24 VMMC providers were trained on VMMC service provision at a cost of US$13,357).

#### Mobile/island outreach sites

The costs of performing VMMC in Goziba Island outreach sites (unique to Kagera) were calculated separately, as there were costs associated with transportation, which include boat rentals. Additionally, there were no health facilities on these hard-to-reach islands and staff had to be transported from the Kagera Regional Hospital to perform the procedure in tents specifically procured for VMMC services.

Table 2 presents the breakdown of direct and indirect costs for VMMC provision in the Goziba Islands in Lake Victoria. Because of high indirect costs, which accounted for the largest share of the unit cost (64%), the cost of delivering one VMMC in the Goziba Islands at US$128.60 was considerably higher than the cost at fixed sites.

### Major Cost Drivers of VMMC in Tanzania

**Figure 1:**
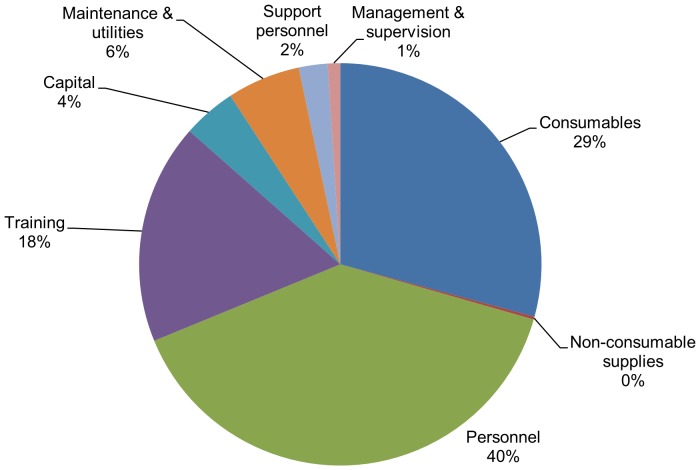
Cost drivers of VMMC in Tanzania, 2010–2011.

Our results show little difference in the costs of campaign and routine models in Tanzania, with unit costs of US$45.38 and US$45.98, respectively. However, the unit cost was found to vary significantly based on the scale of the intervention, with costs being substantially higher in the lowest volume site. Based on our sample, routine service delivery accounted for approximately 60% of the total circumcisions, while campaign service delivery accounted for approximately 39% of all circumcisions. While costs were found to be considerably higher in the mobile/island outreach sites, estimated at US$128.60 per VMMC, in our sample, mobile/island outreach represented only 1% of total circumcisions. By taking an average of the unit costs for each type of service delivery model, weighted by the corresponding percentage of circumcisions performed via each model, our analysis estimates that the average unit cost of performing VMMC in Tanzania (across all service delivery models) was US$46.61. Direct costs accounted for approximately 86.5% of this unit cost, while indirect costs only accounted for approximately 13.5% of the unit cost. Personnel and consumables were the two largest contributors to costs, accounting for 40% and 30% of the total unit cost, respectively (see Figure 1).

### Impact of Scaling Up Voluntary Medical Male Circumcision in Tanzania

#### Total number of male circumcisions

**Figure 2:**
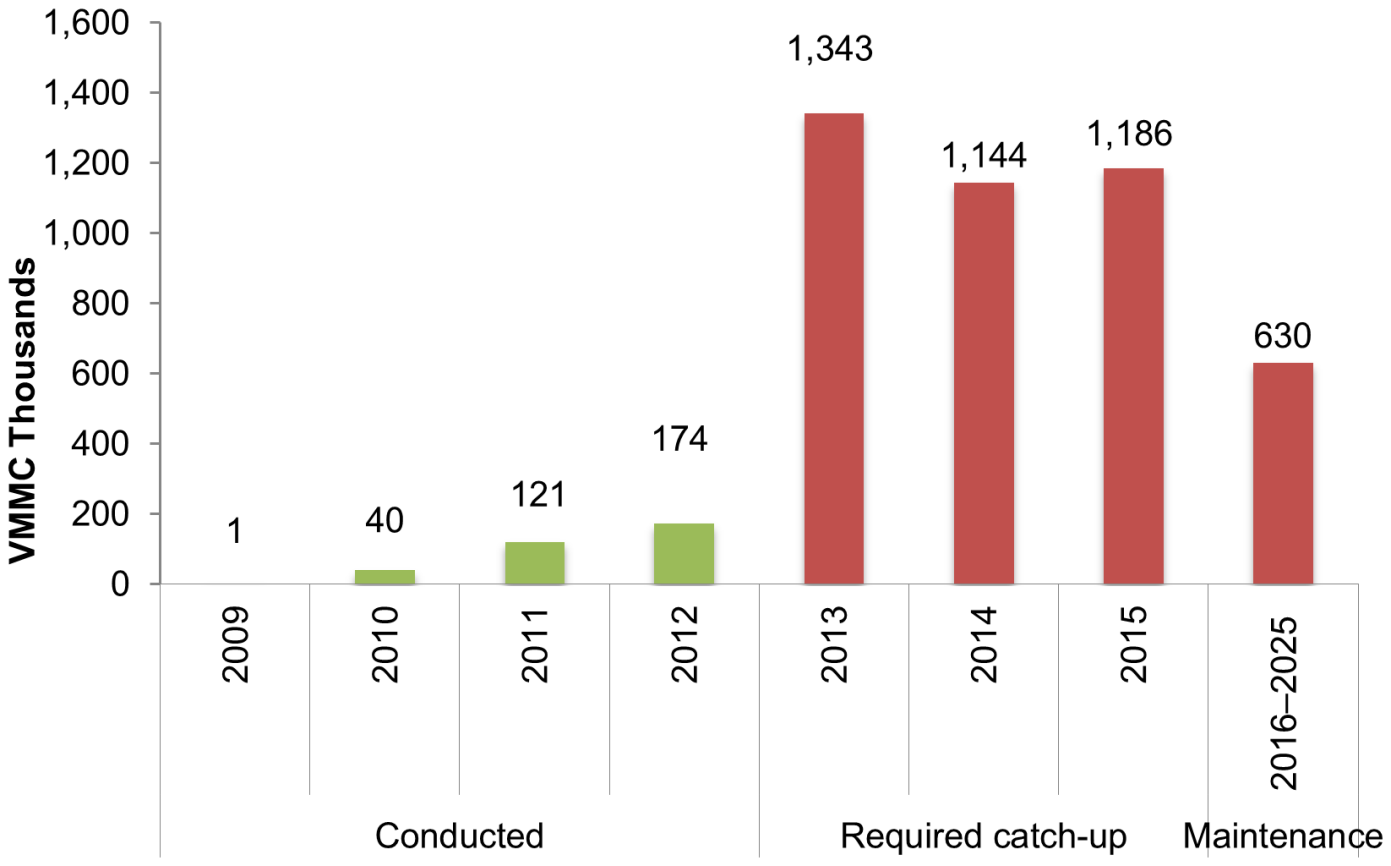
VMMCs conducted (2009–2012) and VMMCs required to catch-up and maintain target (2013–2025).

Figure 2 shows the number of VMMCs conducted from 2009 to 2012, as reported by the Government of Tanzania, as well as the number of annual VMMCs required to reach the target coverage from 2012 to 2025, as estimated by the DMPPT.

At the end of 2012, a total of 335,724 VMMC operations had been completed in Tanzania. A rapid scale-up of VMMC to achieve 87.9% male circumcision prevalence among adult males within a five year “catch-up” period will require a large increase in the number of VMMCs conducted per year in the short term, peaking at about 1.3 million additional circumcisions in 2013 and dropping to about 630,000 circumcisions annually from 2016 to 2025.

### Impact of VMMC Scale-Up on HIV Infection

**Figure 3:**
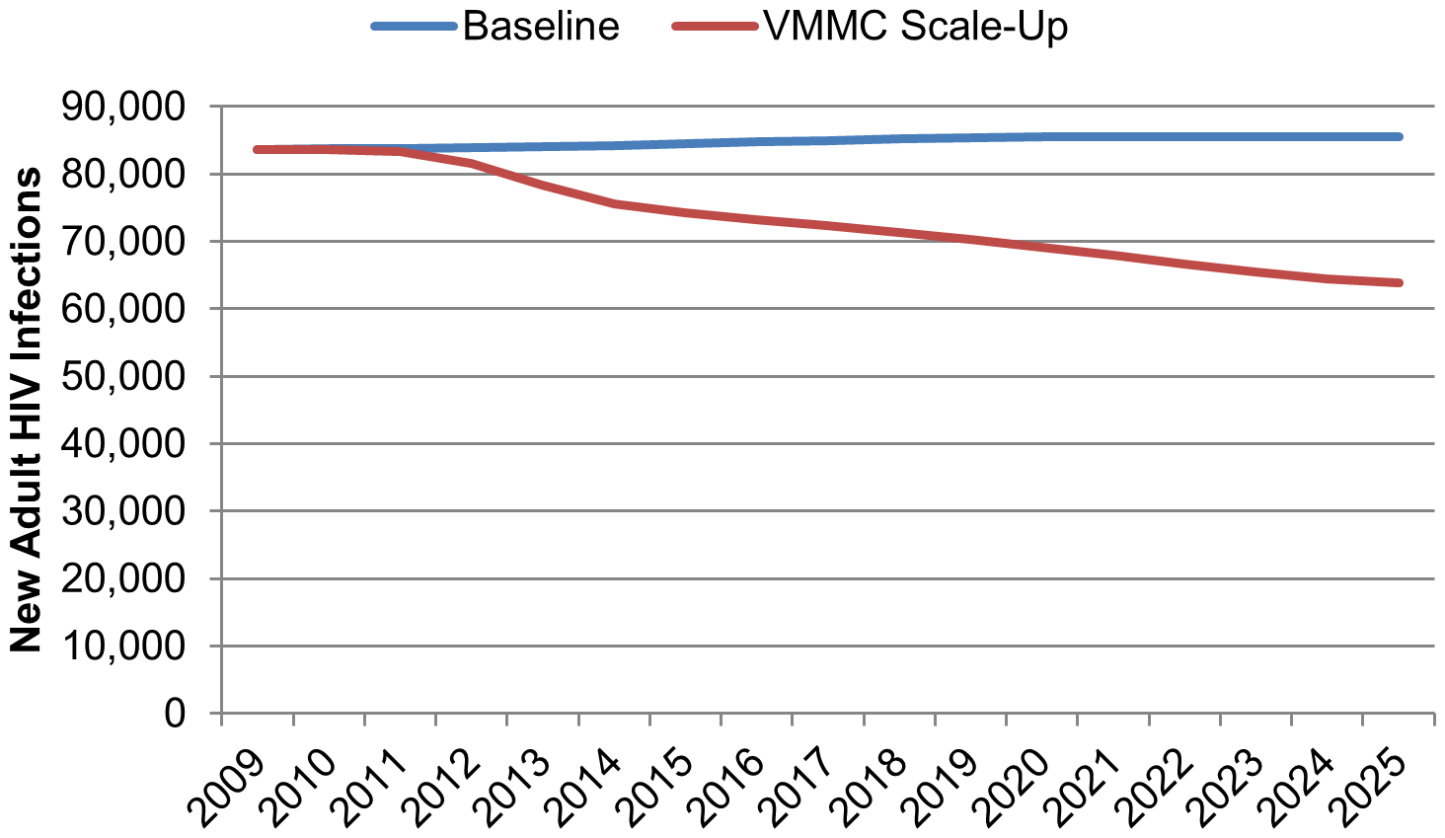
Annual number of new HIV infections baseline and with VMMC scale-up (2009–2025).

With no VMMC scale-up, the annual number of new infections will rise from approximately 84,000 in 2009 to around 85,500 by 2025 (see Figure 3). However, by scaling up VMMC, the number of new HIV infections would rapidly decline, from 84,000 in 2010 to less than 64,000 in 2025. Overall, between 2010 and 2025, a cumulative total of about 190,500 HIV infections, 14% of total new HIV infections, are averted as a result of VMMC scale-up.

With VMMC scale-up, among men ages 10 to 49 years old, the number of new HIV infections is expected to drop by almost half from 38,200 in 2010 to 29,000 in 2025 (as opposed to an increase to 40,700 if current VMMC prevalence persists). Results also indicate that VMMC scale-up will indirectly benefit women, with new HIV infections for women decreasing from about 45,500 in 2010 to 34,900 in 2025. As the risk of HIV infection decreases among men as a direct result of being circumcised, the incidence and prevalence of HIV among men in the population also decreases. Consequently, the likelihood that women will encounter HIV-positive male sex partners decreases.

### Cost Implications of Scaling Up VMMC

**Figure 4:**
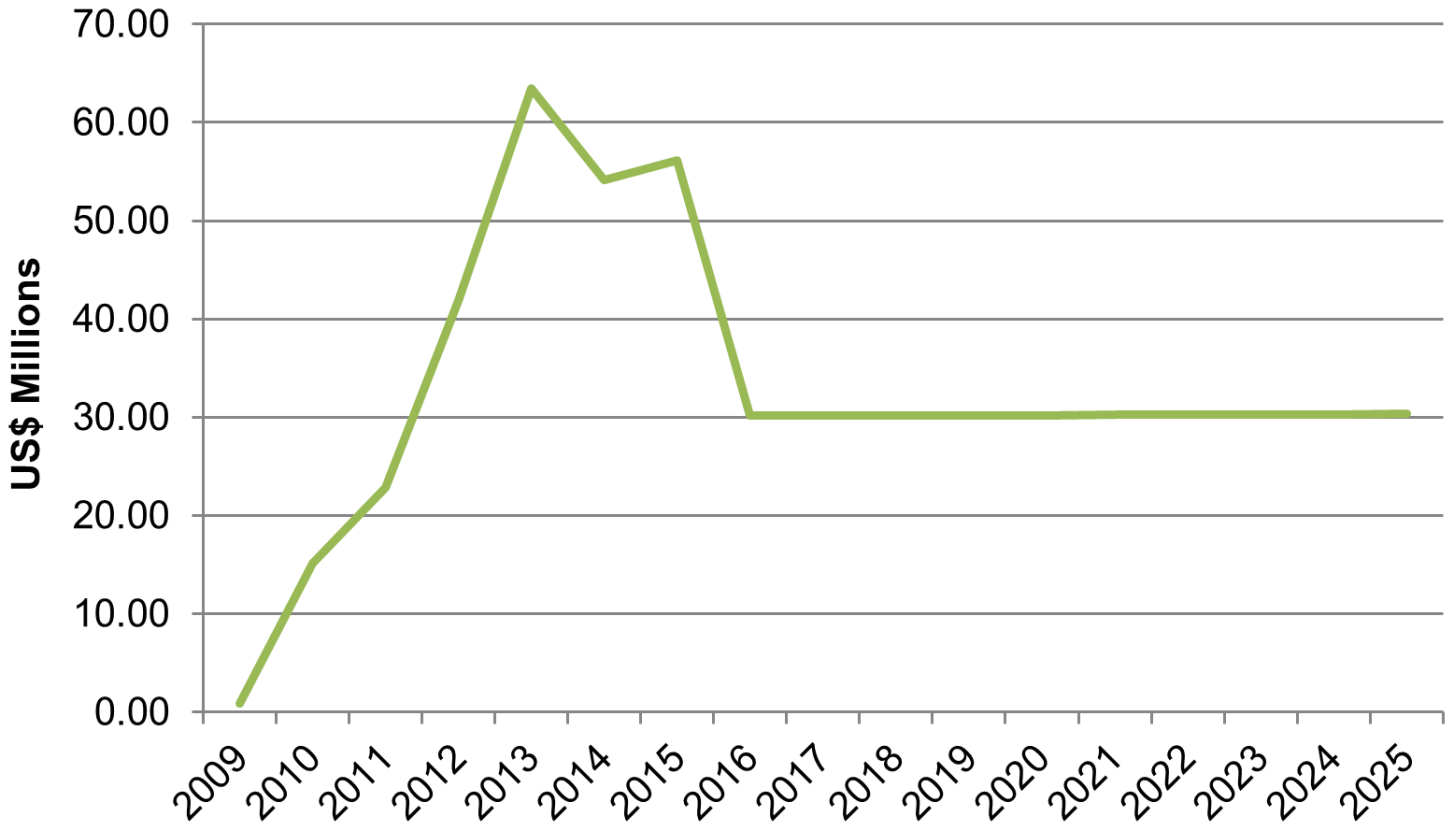
Additional annual cost of scaling up VMMC in Tanzania (2009–2025).

The total cost of scaling up VMMC in the priority regions is high in the short term, peaking in 2013 at about US$63 million/year in additional expenditures above baseline costs (see Figure 4). After the initial intensive scale-up of services through 2015, total annual additional costs decline to about US$30 million in the long term. Between 2010 and 2015, the total additional investment required to achieve the targeted number of VMMCs is estimated at US$253.7 million, while US$302.4 million would be required from 2016 to 2025.

### Cumulative Costs and Cost Savings

**Figure 5:**
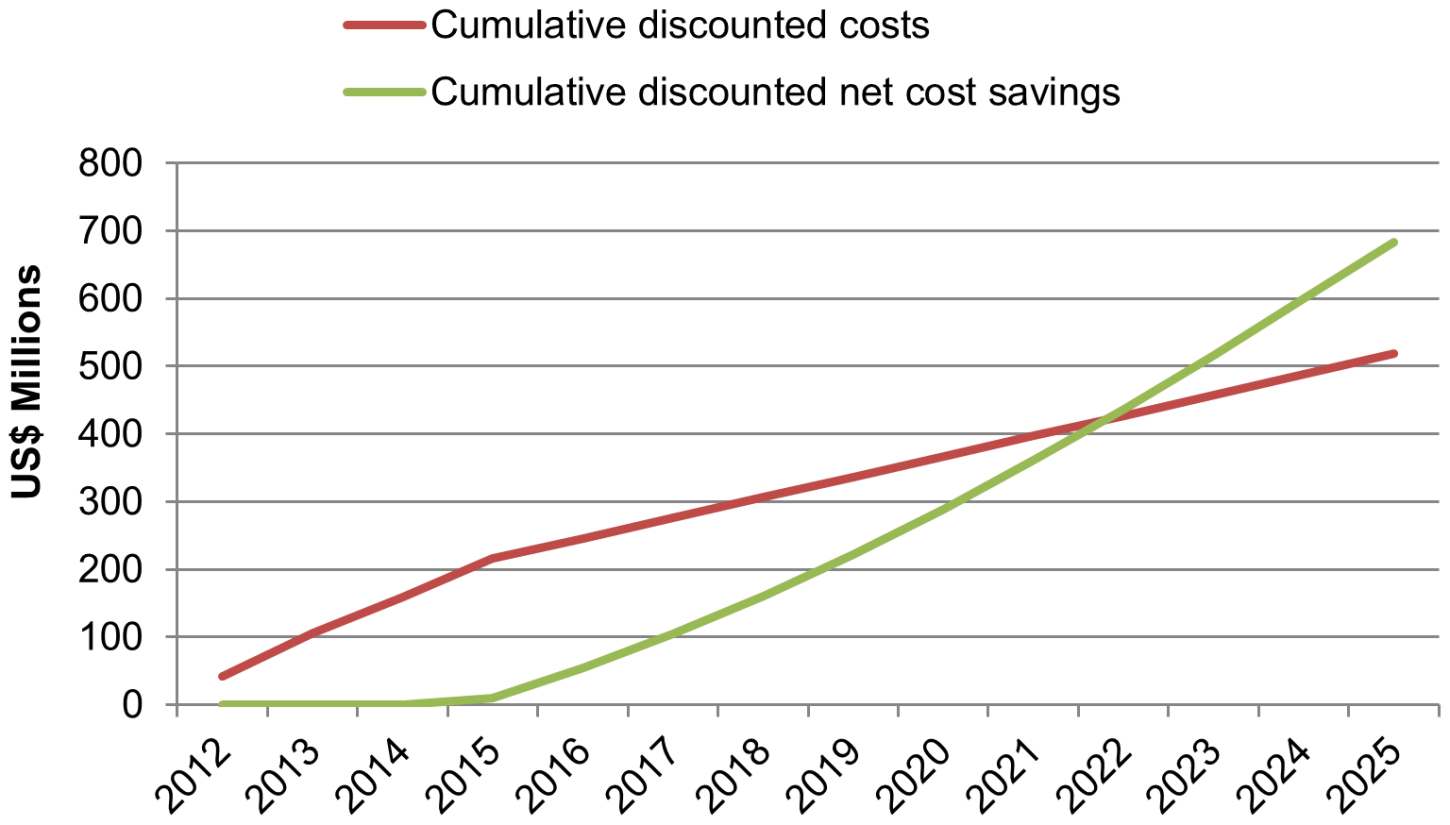
Cumulative net costs and cumulative net savings with VMMC scale-up (2010–2025).

As shown in Figure 5, the cumulative discounted costs (the cumulative costs associated with scaling up VMMC) are projected to rise rapidly until 2015, and then to increase more gradually thereafter. The cumulative discounted benefits (the value of averting future treatment costs) rise more gradually in the first few years, but then rapidly accumulate starting around 2015. By 2022, the discounted cumulative benefits exceed the discounted cumulative costs. As projections go further into the future, the full financial benefits of preventing an HIV infection are observed.

### Study Limitations

For several reasons, the results from this analysis may not be fully representative of the costs associated with scaling up male circumcision across Tanzania. First, the sample of sites was relatively limited. A more comprehensive costing of sites might produce different results.

Second, a number of costs, including demand creation and waste management, were excluded from the unit cost analysis because of lack of available data. Additionally, the analysis used the global default value for lifetime discounted antiretroviral treatment costs (US$7,500), due to lack of country-specific information. If Tanzania pays less for treatment than this global default indicates, the financial savings associated with male circumcision would be less due to an overestimation of treatment costs. Conversely, if the lifetime cost of treatment is higher in Tanzania than this global value suggests, the savings could be higher than indicated in this study.

Finally, since the time of data collection (2010/2011) was relatively early in Tanzania's VMMC scale-up, the costs associated with this study may not be representative of current scale-up costs. The VMMC program in Tanzania has not only expanded significantly, but various elements of the program have changed as well. As programs target “late adopters,” it is possible that significantly more resources may be needed to reach these populations. Conversely, it is possible that as programs become more routinized and high levels of scale-up are achieved at each facility, economies of scale may take effect, bringing down unit costs. While economies of scale are expected as services are scaled up, diseconomies of scale are possible at later stages of the program, as greater resources are required to reach the remaining, “hard-to-reach” populations.

## Discussion

The results of this analysis show that direct costs accounted for the vast majority (86.5%) of the average unit cost of US$46 for VMMC in Tanzania. This unit cost estimate is consistent with values reported from VMMC costing studies in Kenya, Uganda, Zambia, and South Africa, which range from US$31.84 in Kenya to US$100.07 in Zimbabwe [9]. Personnel and consumables were the largest drivers of unit cost of VMMC in Tanzania and accounted for 40 and 30% of the unit costs, respectively.

The study results show that increasing the prevalence of male circumcision from current levels to 87.9% by 2015 and maintaining this coverage thereafter will require an additional cost of US$253.7 million through 2015 and US$302.3 million between 2016 and 2025, with an average cost per HIV infection averted of US$3,200 between 2010 and 2025. Scaling up VMMC will require increasing the annual number of circumcision procedures to about 1.3 million in 2013 before leveling off to around 630,000 after 2015.

Although initial intensive scale-up of services will require a large investment in the early years, this scale-up would avert nearly 23,000 new adult HIV infections through 2015 and almost 167,500 between 2016 and 2025. A successful VMMC program in Tanzania can yield discounted net savings of around US$4,200 per infection averted during 2010–2025.

Given the health and economic benefits of investing in VMMC, it is crucial that the Government of Tanzania, development partners, and relevant stakeholders leverage funds and mobilize resources for rapid and full VMMC scale-up as a central component of the national HIV prevention strategy in Tanzania. To meet the coverage target in a short timeframe and achieve the projected health impact and cost savings, there must be a strong focus on improving VMMC program efficiencies by matching supply with existing demand. This can be achieved by ensuring the retention of dedicated human resources, reducing labor costs through task shifting and task sharing, improving supply chain systems, and decreasing the cost of commodities, while also simultaneously creating demand through targeted communication in areas with low demand, guaranteeing access to services by providing dedicated space, and using mobile and outreach sites to bring services to the Tanzanian population.
